# Pre/post evaluation of a pilot prevention with positives training program for healthcare providers in North West Province, Republic of South Africa

**DOI:** 10.1186/s12913-017-2263-7

**Published:** 2017-05-02

**Authors:** Christopher G. Kemp, Julia de Kadt, Erushka Pillay, Jennifer M. Gilvydis, Evasen Naidoo, Jessica Grignon, Marcia R. Weaver

**Affiliations:** 1 Department of Global Health, University of Washington, Ninth and Jefferson Building, 13th Floor, Box 359932, 908 Jefferson Street, Seattle, WA 98104 USA; 2 International Training and Education Center for Health (I-TECH), University of Washington, 908 Jefferson Street, Seattle, WA 98104 USA; 3 I-TECH South Africa, 232 Bronkhorst St, Suite 203 Optiplan House, Nieuw Muckleneuk, Pretoria, South Africa; 4 Project Hope, 255 Carter Hall Lane, PO Box 250, Millwood, VA 22646 USA

**Keywords:** HIV/AIDS, Prevention with positives, Positive health, Dignity and prevention, Audio-computer assisted structured interviews, Evaluation, South Africa

## Abstract

**Background:**

Prevention interventions for people living with HIV/AIDS are an important component of HIV programs. We report the results of a pilot evaluation of a four-hour, clinic-based training for healthcare providers in South Africa on HIV prevention assessments and messages. This pre/post pilot evaluation examined whether the training was associated with providers delivering more prevention messages.

**Methods:**

Seventy providers were trained at four public primary care clinics with a high volume of HIV patients. Pre- and post-training patient exit surveys were conducted using Audio-Computer Assisted Structured Interviews. Seven provider appropriate messaging outcomes and one summary provider outcome were compared pre- and post-training using Poisson regression.

**Results:**

Four hundred fifty-nine patients pre-training and 405 post-training with known HIV status were interviewed, including 175 and 176 HIV positive patients respectively. Among HIV positive patients, delivery of all appropriate messages by providers declined post-training. The summary outcome decreased from 56 to 50%; adjusted rate ratio 0.92 (95% CI = 0.87–0.97). Sensitivity analyses adjusting for training coverage and time since training detected fewer declines. Among HIV negative patients the summary score was stable at 32% pre- and post-training; adjusted rate ratio 1.05 (95% CI = 0.98–1.12).

**Conclusions:**

Surprisingly, this training was associated with a decrease in prevention messages delivered to HIV positive patients by providers. Limited training coverage and delays between training and post-training survey may partially account for this apparent decrease. A more targeted approach to prevention messages may be more effective.

**Electronic supplementary material:**

The online version of this article (doi:10.1186/s12913-017-2263-7) contains supplementary material, which is available to authorized users.

## Background

Prevention interventions for people living with HIV/AIDS (PLHIV), known as “Prevention with Positives” (PWP), are an important component of comprehensive HIV programs. Recently, the President’s Emergency Plan for AIDS Relief (PEPFAR) defined a minimum package of PWP interventions that included five assessments and provision of condoms and lubricants at all healthcare encounters. Assessments were recommended on: 1) patients’ adherence to anti-retroviral therapy (ART), 2) signs and symptoms of sexually transmitted infections (STI) and other opportunistic infections, 3) partners’ status and disclosure, 4) reproductive health/family planning intentions, and 5) patients’ sexual risk-behavior, and alcohol and other substance use [1]. A subsequent systematic review reported that PWP interventions can bolster the impact of treatment programs on reducing morbidity and HIV transmission in low- and middle-income countries [2].

PWP interventions are particularly relevant in the Republic of South Africa, where an estimated 6.43 million people are living with HIV/AIDS, and HIV prevalence is 29.5% among pregnant women aged 15–49 attending antenatal clinics [3–5]. As people with HIV live longer with improved treatment, they need knowledge and support to protect their own health and avoid transmitting HIV to others [6]. One of the sub-objectives of South Africa’s National Strategic Plan for HIV, STI, and Tuberculosis is to “increase access to a package of sexual and reproductive health services” for PLHIV and young people [7].

Integrated prevention interventions that are delivered in healthcare settings and address medical, social and psychological factors have been shown to be particularly effective [8–10]. In the United States, there is evidence that provider-delivered PWP interventions significantly increase the number of prevention conversations, reduce the mean number of sexual partners, and decrease the prevalence of unprotected intercourse [11, 12]. A review of PWP trials concluded that the duration of effects of provider-delivered interventions is longer than that of other interventions [13]. Although it is clearly crucial that providers in South Africa be similarly trained to deliver PWP interventions, there is a lack of evidence of the effectiveness of training packages designed to support providers to deliver them in this context.

The United States Centers for Disease Control and Prevention (CDC), with support from the University of Washington’s International Training and Education Center for Health (I-TECH), developed a four-hour, clinic-based training to equip healthcare providers with standard HIV-prevention assessment questions and messages, adapted from *Partnership for Health* for South Africa [14]. The training introduces a job aid, “*Five HIV Prevention Steps for People Living with HIV/AIDS: A Tool for Healthcare Providers,”* with assessment questions and prevention messages for HIV positive patients.

The results of a pre/post evaluation conducted by I-TECH of a pilot training at public-sector primary healthcare (PHC) clinics in the North West Province of South Africa are reported below. We tested whether this training intervention increased patient-reported receipt of HIV-prevention messages during routine patient care.

## Methods

### PWP training intervention

Nurses and counselors were trained at four PHC clinics serving high volumes of patients receiving pre-ART and ART services in Bojanala Platinum District of North West Province. Clinic selection was conducted in collaboration with the District Management Team of the Department of Health. HIV prevalence in the district was 35.0% among pregnant women aged 15–49 attending antenatal clinics, compared to 29.5% nationally as noted above [4]. HIV fatalism, restrictive gender norms, HIV-related stigma, and general discomfort communicating about sex have been identified at the community and clinic levels in the district, and hamper progress in prevention and treatment [15].

The three training objectives were: 1) describe the importance of making recommendations to HIV positive patients about HIV prevention; 2) explain the importance of each step that makes up a comprehensive strategy for addressing prevention with HIV positive individuals; and 3) demonstrate use of the provider job aid for delivering key prevention recommendations and strategies to HIV positive patients.

The training focused on the *Five HIV Prevention Steps for People Living with HIV/AIDS: A Tool for Health Care Providers*. This job aid is presented in Additional file 1. Providers were taught to assess for risk and deliver prevention messages according to five themes in the job aid: 1) prevention recommendations on sexual behavior, safer sex, and alcohol and substance use, 2) adherence to ART and other medications, 3) signs and symptoms of STIs, 4) pregnancy status and intentions, and 5) condom demonstration.

I-TECH worked with clinic managers to develop a training implementation plan. The curriculum was the same in all four facilities and the same trainers conducted all sessions. Fidelity of trainings to methods and content were similar across facilities and sessions. The four-hour training session was delivered several times over one to four months, with a range of two to four training dates per clinic. All nurses at two of the clinics were trained (19 of 19 at clinic B, and 10 of 10 at clinic D), 90% at clinic A (9 of 10), and 66% at clinic C (14 of 21). 18 counsellors and other staff across the four clinics were also trained. As part of the training session, providers developed personal action plans for conducting the assessments and delivering the messages. Following the trainings, copies of the action plans were given to the providers and to a training coordinator. The coordinator contacted each trained provider by phone, every two weeks for eight total weeks, to review progress on and document revisions or updates to the action plans.

### Design

We used patient exit surveys to collect data from a convenience sample of HIV positive patients pre- and post-training at four clinics. This survey was designed to avoid revealing the patients’ HIV status to the data collection team or to others. Patients responded to all questions anonymously via Audio Computer Assisted Self Interview (ACASI), including to the question about HIV status. ACASI has been shown to be a feasible and reliable data collection method in South Africa [16–19]. To avoid disclosure through participation, all patients were invited to complete the questionnaire, regardless of HIV status. All patients were asked about HIV prevention assessments conducted by the provider, and messages from the provider, though HIV negative patients skipped questions that were only appropriate for patients with HIV. Data from HIV negative patients served as a control for other pre/post secular trends and changes in provider behavior at these clinics. Patients were eligible to participate if they received care at the clinic on the date of survey, were aged 18 and above, and were fluent in English or Setswana.

Survey questions about HIV prevention assessments and messages varied across respondents depending on their self-reported risk behaviors and HIV status. For example, only patients who reported being sexually active in the previous three months were asked whether their provider discussed the HIV status of their sexual partner(s). The exit interview questions for HIV positive patients are presented in Additional file 2.

Eligible participants gave informed consent and received instruction on using an electronic tablet. The ACASI questionnaire was designed with the Open Data Kit and data were uploaded onto FormHub© each evening [20, 21]. The pre-test survey was conducted from December 2013 to February 2014, and the post-test was conducted from August to December 2014.

Sample-size calculations assumed that training would be associated with a 20% absolute increase in appropriate messaging among HIV positive patients, giving a sample size of 140 per time period for a two-sample test of proportions with a power of 0.90 and alpha of 0.05. This was increased to 175 to allow for 25% incomplete or missing data. Patient interviews continued until 175 HIV positive patients were interviewed during each time period.

### Ethical considerations

The evaluation protocol was reviewed and approved by the North West Province Department of Health Research Committee and the Human Sciences Research Council of South Africa (REC 8/20/02/13). The Human Subjects Division at the University of Washington determined that the evaluation did not meet the regulatory definition of research under 45 CFR 46.102(d).

Informed consent was obtained from all interview participants. No identifying information was collected. Participants sat where no one could observe their answers, used headphones, and answered questions by tapping a tablet screen. Participants did not receive compensation.

### Data analysis

Seven outcome variables were generated to measure provider messaging. Three were derived from the prevention theme of the PWP training package: sexual activity, safer sex, and drugs and alcohol. One outcome variable was generated for each of four remaining themes of the intervention: adherence to ART and other medications, signs and symptoms of STIs, pregnancy status and intentions, and condom demonstration. These were summed to calculate a summary provider messaging score.

Poisson regression was conducted with the patient as the unit of analysis. The outcome variables were the number of appropriate messages received, adjusting for the number of potential messages that should be delivered as the exposure. The number of potential messages varied by patients’ HIV status, behaviors, and responses to assessment questions. Skipped questions, as noted in Additional file 2, were not included in the denominator. Models adjusted for time period, HIV status, the interaction of the two, and seven additional potential confounders chosen *a priori*: 1) clinic, 2) profession of healthcare provider (nurse, counselor, doctor, other) 3) patient age (18–29, 30–39, 40–49, 50+), 4) gender, 5) education (no schooling/some primary/completed primary, some secondary or completed secondary, college/university/technical, or other, 6) race, and 7) language. All analyses used robust standard errors to adjust for over-dispersion, and were performed in Stata 13 [22].

The primary analyses were performed with complete cases. Those with missing data on provider assessment or messaging were excluded based on the assumption of data missing at random. Three sensitivity analyses were conducted to test alternative assumptions regarding missing data: 1) provider did not deliver the message, 2) provider did deliver the message, and 3) missing message could be excluded from the denominator.

Three additional sensitivity analyses were conducted. The first focused on nurses and counselors, excluding patients who saw doctors (*n* = 96) and other providers (*n* = 12), because nurses and counsellors were the intended audience for the training. The second focused on the three clinics with relatively high training coverage, excluding Clinic C (*n* = 332) at which only 14 of 21 nurses were trained. The third adjusted for the delay between training and post-training data collection among HIV positive patients. Observations were analyzed by quartiles from the shortest to the longest delay.

## Results

**Fig. 1:**
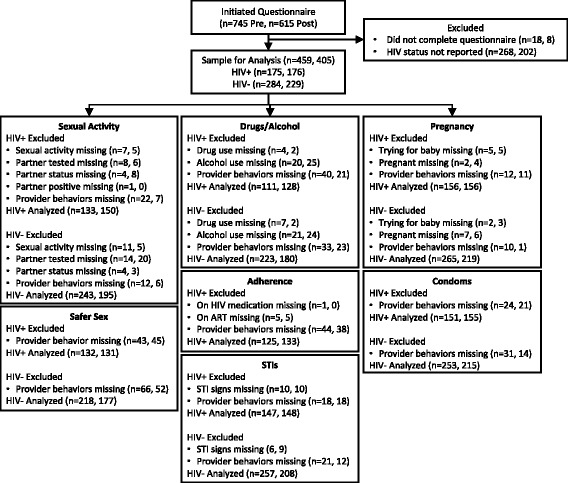
Sample inclusion and exclusion pre- and post-training by outcome

Seven hundred forty-five patients initiated the interview at pre-training and 615 at post-training. Figure 1 documents sample sizes for each outcome pre- and post-training. After excluding individuals who did not report their HIV status, 459 observations remained at pre-training and 405 at post-training. These included 175 HIV positive patients pre-training and 176 HIV positive patients post-training.

Descriptive statistics on the included patient samples are presented in Table 1, stratified by HIV status and time period. More than 80% of patient visits were with nurses, with some patients seeing more than one provider in a single clinic visit. There were some differences between samples pre- and post-training. Pre-training, 63% of HIV positive patients and 46% of HIV negative patients chose to answer the questionnaire in Setswana, compared to 34 and 20% respectively post-training. Similarly, 25% of HIV positive patients and 55% of HIV negative patients fell into the 18–29 age range pre-training, compared to 34 and 72% respectively post-training.

**Table 1:** Descriptive statistics of subjects by HIV status and time period

		HIV Positive		HIV Negative	
	Missing	Pre	Post	Pre	Post
N		175	176	284	229
Provider Characteristics					
Facility	0				
A		46 (26.3%)	42 (23.9%)	73 (25.7%)	48 (21.0%)
B		24 (13.7%)	23 (13.1%)	75 (26.4%)	61 (26.6%)
C		80 (45.7%)	76 (43.2%)	103 (36.3%)	73 (31.9%)
D		25 (14.3%)	35 (19.9%)	33 (11.6%)	47 (20.5%)
Provider	0				
Nurse		147 (84.0%)	161 (91.5%)	251 (88.4%)	206 (90.0%)
Counselor		23 (13.1%)	14 (8.0%)	32 (11.3%)	22 (9.6%)
Doctor		26 (14.9%)	15 (8.5%)	34 (12.0%)	21 (9.2%)
Other		2 (1.1%)	1 (0.6%)	8 (2.8%)	1 (0.4%)
Client Characteristics					
Gender	0				
Male		46 (26.3%)	32 (18.2%)	51 (18.0%)	35 (15.3%)
Female		129 (73.7%)	144 (81.8%)	233 (82.0%)	194 (84.7%)
Age	0				
18–29		43 (24.6%)	60 (34.1%)	155 (54.6%)	166 (72.5%)
30–39		64 (36.6%)	76 (43.2%)	58 (20.4%)	37 (16.2%)
40–49		46 (26.3%)	29 (16.5%)	33 (11.6%)	13 (5.7%)
50+		22 (12.6%)	11 (6.3%)	38 (13.4%)	13 (5.7%)
Education	0				
None, some or completed primary		26 (14.9%)	16 (9.1%)	27 (9.5%)	12 (5.2%)
Some or completed secondary		125 (71.4%)	130 (73.9%)	176 (62.0%)	151 (66.0%)
College, University or Technikon		11 (6.3%)	19 (10.8%)	66 (23.2%)	59 (25.8%)
Other (e.g., ABET)		13 (7.4%)	11 (6.3%)	15 (5.3%)	7 (3.1%)
Race	0				
Black		169 (96.6%)	170 (96.6%)	268 (94.4%)	224 (97.8%)
Other		6 (3.4%)	6 (3.4%)	16 (5.6%)	5 (2.2%)
Language	0				
English		65 (37.1%)	117 (66.5%)	152 (53.5%)	184 (80.4%)
Setswana		110 (62.9%)	59 (33.5%)	132 (46.5%)	45 (19.7%)
Sexually active within last 3 months	0				
Yes		92 (52.6%)	92 (52.3%)	168 (59.2%)	144 (62.9%)
No		76 (43.4%)	79 (44.9%)	105 (37.0%)	80 (34.9%)
Decline		5 (2.9%)	2 (1.1%)	7 (2.5%)	2 (0.9%)
Do not know		2 (1.1%)	3 (1.7%)	4 (1.4%)	3 (1.3%)
Years since HIV diagnosis	63				
Less than 1 year		76 (43.9%)	42 (36.5%)		
1–2 years		19 (11.0%)	12 (10.4%)		
> 2 years		78 (45.1%)	61 (53.0%)		
Have children	167				
Yes		79 (85.9%)	80 (87.0%)		
No		12 (13.0%)	12 (13.0%)		
Do not know		1 (1.1%)	0 (0%)		
Currently on ART	55				
Yes		127 (87.6%)	134 (88.7%)		
No		13 (9.0%)	12 (8.0%)		
Decline		1 (0.7%)	2 (1.3%)		
Do not know		4 (2.8%)	3 (2.0%)		

Among HIV positive patients, the percentages who were women (73.7% pre-training and 81.8% post-training) and enrolled in ART (87.6% pre-training and 88.7% post-training) were higher than nationally (60.0 and 31.2%, respectively) [3]. This would be expected from a facility-based sample, given that nationally the percentage of women who have initiated ART is higher (34.7%) than men (25.7%), and patients on ART have regular clinic visits [3]. The percentages that were Black (96.6% pre-training and 94.4% post-training) were similar to the national percentage (97.3%) [3].

### Appropriate provider messaging

**Fig. 2:**
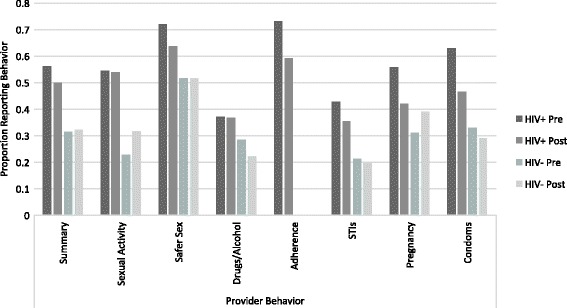
Average proportion of appropriate messages by outcome and time period

Among HIV positive patients, mean percentage of appropriate messages reported was lower post-training for all seven outcomes. The summary score decreased from 56% pre-training to 50% post-training. Among HIV negative patients, the mean percentages increased post-training for sexual activity and pregnancy status and intentions, but decreased for drugs and alcohol, and signs and symptoms of STIs. The summary score for HIV negative patients was 32% at both time periods (Fig. 2).

Table 2 reports the regression results. Among HIV positive patients, the proportion of appropriate messages delivered was lower post-training than pre-training. For the total score, the adjusted rate ratio was 0.92 (95% CI = 0.87–0.97). Rate ratios were less than 1 for six of the seven outcomes, and statistically significant for three outcomes: 1) adherence to ART or other medications, 2) pregnancy status and intentions, and 3) condom distribution. The exception was drugs and alcohol, for which the proportion increased post-training, but the increase was not statistically significant; adjusted rate ratio 1.13 (95% CI = 0.94–1.36).

**Table 2:** Rate ratios for appropriate prevention messaging from Poisson models

		HIV Positive			HIV Negative			Difference in Difference		
Outcome	Analysis	RR	95% CI	*p*-value	RR	95% CI	*p*-value	Ratio of RR	95% CI	*p*-value
Summary	Unadjusted	0.87	0.82–0.91	<0.001	1	0.94–1.07	0.943	0.86	0.80–0.94	0.001
	Adjusted	0.92	0.87–0.97	0.004	1.05	0.98–1.12	0.133	0.88	0.81–0.95	0.002
	*n* = 864									
Sexual activity	Unadjusted	0.83	0.70–0.99	0.038	1.33	1.06–1.67	0.014	0.63	0.47–0.83	0.001
	Adjusted	0.99	0.82–1.19	0.901	1.52	1.20–1.93	0.001	0.65	0.49–0.87	0.003
	*n* = 721									
Safer sex	Unadjusted	0.88	0.79–0.99	0.031	1	0.89–1.12	0.997	0.88	0.76–1.04	0.129
	Adjusted	0.92	0.82–1.03	0.159	1.03	0.91–1.16	0.637	0.89	0.76–1.05	0.167
	*n* = 658									
Adherence to ARVs and other meds	Unadjusted	0.83	0.73–0.95	0.006						
	Adjusted	0.85	0.74–0.98	0.023						
	*n* = 258									
Drug and alcohol	Unadjusted	0.99	0.83–1.18	0.927	0.77	0.63–0.96	0.017	1.28	0.98–1.68	0.074
	Adjusted	1.13	0.94–1.36	0.185	0.85	0.68–1.06	0.149	1.33	1.01–1.75	0.042
	*n* = 642									
Signs and symptoms of STIs	Unadjusted	0.82	0.70–0.95	0.01	0.96	0.80–1.15	0.643	0.85	0.67–1.08	0.178
	Adjusted	0.89	0.75–1.05	0.163	1.04	0.86–1.25	0.705	0.86	0.68–1.09	0.209
	*n* = 760									
Pregnancy status and intentions	Unadjusted	0.78	0.65–0.93	0.005	1.22	0.97–1.55	0.093	0.63	0.47–0.85	0.002
	Adjusted	0.75	0.62–0.90	0.002	1.16	0.91–1.48	0.24	0.65	0.48–0.87	0.004
	*n* = 596									
Condom Demonstration	Unadjusted	0.74	0.66–0.83	<0.001	0.89	0.79–1.00	0.053	0.83	0.71–0.98	0.027
	Adjusted	0.81	0.72–0.91	0.001	0.95	0.84–1.08	0.437	0.85	0.72–1.01	0.058
	*n* = 774									

*RR* Rate ratio, *CI* Confidence interval

Among HIV negative patients, the proportion of appropriate messages delivered was generally the same pre- and post-training. For the total score, the adjusted rate ratio was 1.05 (95% CI = 0.98–1.12). For sexual activity, the proportion of appropriate messages increased significantly post-training; adjusted rate ratio 1.52 (95% CI = 1.20–1.93).

We failed to reject the null hypothesis that the effect of the training program was equivalent among HIV positive and HIV negative patients. In the difference-in-difference analysis, the effect was smaller and negative among people who were HIV positive. For the total score, the adjusted ratio of rate ratios was 0.89 (95% CI = 0.81–0.95). The exception was drugs and alcohol, for which the adjusted ratio of rate ratios was 1.33 (95% CI = 1.01–1.75).

Results of the sensitivity analyses were substantially the same with regards to alternative assumptions for missing values and when excluding patients who saw providers other than nurses or counselors. However, results for the sensitivity analysis among the three clinics with relatively high training coverage (excluding Clinic C) differed substantially from the primary analysis. Among HIV positive patients, the total score post-training was the same as pre-training; adjusted rate ratio 1.00 (95% CI = 0.92, 1.07). The proportion of drug and alcohol messages increased significantly post-training; adjusted rate ratio 1.33 (95% CI = 1.03, 1.71), and the proportion of pregnancy status and intention messages decreased significantly; adjusted rate ratio 0.77 (95% CI = 0.60, 1.00). Results of the sensitivity analysis of the delay between training and post-test data collection also differed from the primary analysis. Results for all outcomes were more favorable among HIV positive patients interviewed immediately post-training. The summary score post-training was similar to pre-training in the first quartile of time delay; adjusted rate ratio 0.95 (95% CI = 0.87, 1.04). As the delay increased, training was associated with a decrease in the proportion of appropriate messages for the summary score and six of the seven outcomes.

## Discussion

This evaluation reveals that the clinic-based PWP training was associated with a significant decrease in prevention messaging for HIV positive patients in the adjusted multivariate analysis, as measured by the summary of patient reports. This decrease was consistent across six of the seven primary outcomes, and statistically significant in three. Results of the sensitivity analyses indicate that some of the decrease in prevention messages after training may be attributed to one clinic with low training coverage, and to delays between training and post-test data collection.

The training emphasized asking all assessment questions and delivering all prevention messages at every patient encounter, alongside the importance of repetition in motivating change in patients’ behaviors. However, clinicians may be reluctant to repeat themselves. They may have asked some of the assessment questions and delivered some of the prevention messages immediately after the training, but did not repeat the questions or messages at later visits. In Kenya, nurses reported an increase in their assessments and messages immediately after training on PWP, and a subsequent decrease two months post-training [23].

A more targeted approach to prevention messages might be more effective and sustainable. ACASI has been used at sexual health clinics to conduct assessments [24–26]. Clinicians could deliver targeted messages based on assessment results, and prioritize concerns and devote time to a meaningful dialogue about one or two risk behaviors and risk-reduction strategies. Alternatively, an ACASI assessment could be coupled with computer-based counseling. In a recent trial in the United States, HIV positive patients in one arm received an ACASI assessment, computerized counseling, and standard clinic visit with a healthcare provider, and those in the other received no computerized counseling [27]. Computerized counseling was associated with lower HIV transmission risk-behaviors than assessment by ACASI and standard of care.

The PWP training program and evaluation had several strengths. The job aid was adapted for South Africa from an evidence-based intervention. Clinic-based training improved access to the training sessions and reduced the time that healthcare providers spent traveling to training sessions. The ACASI questionnaire focused on clinician messaging to minimize respondent burden. Audio recordings were available in two languages and expertly engineered. Sensitivity analyses indicated a positive effect on provider behavior regarding alcohol and drug messaging in the clinics with high training coverage; communities in this district consider alcohol abuse to be a significant problem [15].

This evaluation had several limitations. Convenience sampling of patients, along with the lack of pre- and post-training data collection on the same patients, meant that the samples were not the same across surveys. However, we adjusted for observed patient characteristics that may have differed pre- to post-training. The lack of an HIV positive control group restricts our ability to draw causal inference or account for secular trends that may have only affected HIV positive patients. However, the samples of HIV negative patients controlled for secular trends that affected all patients. Our limited timeframe prevented a pre-test of the questionnaire with a smaller sample of patients. Issues with the skip pattern for the sexual activity questions were not identified until after pre-training data were collected and analyzed, meaning some data on disclosure of HIV status and HIV testing for children could not be analyzed. Post-training data collection occurred up to eight months after-training, and may have missed the immediate effects of the training program. The survey did not distinguish between patients who saw trained providers from those who saw untrained providers.

## Conclusions

Contrary to expectations, the clinic-based PWP training was associated with a decrease in prevention messages delivered to HIV positive patients by healthcare providers. Limited training coverage may have contributed to this overall decrease. Moreover, the delay between training and post-training interviews may have allowed the pilot evaluation to miss the short-term impact of the training. A more targeted approach to prevention messages may be more effective.

## Additional files

Additional file 1: Prevention with Positives Job Aid. This job aid, titled “Five HIV Prevention Steps for People Living with HIV/AIDS: A Tool for Health Care Providers,” helps providers target appropriate prevention messages for their patients living with HIV/AIDS. (PDF 229 kb)

Additional file 2: HIV Positive Patient Exit Questionnaire. This is the questionnaire that was designed for our study and completed by HIV positive patients at health facilities. (DOCX 41 kb)

## Abbreviations

ACASI: Audio computer assisted self interview
ART: Antiretroviral therapy
CDC: Centers for disease control and prevention
I-TECH: International training and education center for health
PEPFAR: President’s emergency plan for AIDS relief
PHC: Primary healthcare
PLHIV: People living with HIV/AIDS
PWP: Prevention with positives
STI: Sexually transmitted infections
