# Supplementary material for: Pre/post evaluation of a pilot prevention with positives training program for healthcare providers in North West Province, Republic of South Africa
Source: BMC Health Serv Res. 2017 May 2;17:316. doi: 10.1186/s12913-017-2263-7 (PMC5414361; doi:10.1186/s12913-017-2263-7)
Supplement: Supplementary file 1 — Prevention with Positives Job Aid. This job aid, titled “Five HIV Prevention Steps for People Living with HIV/AIDS: A Tool for Health Care Providers,” helps providers target appropriate prevention messages for their patients living with HIV/AIDS. (PDF 229 kb) [file 12913_2017_2263_MOESM1_ESM.pdf]

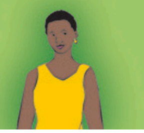

Five HIV Prevention Steps for People Living with HIV/AIDS

A Tool for Health Care Providers

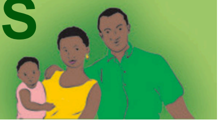

Deliver all five prevention steps at every visit. Help patients choose a prevention goal to address based on their circumstances and discuss during the visit.

STEP 1: GIVE prevention recommendations to your HIV patient.

<u>ASSESS</u> client's sexual activity, and partner(s)' and children's HIV status.

⏩ Tell me about your sexual activity.

If SEXUALLY ACTIVE:

<u>INFORM</u> all sexually active patients:

- Your sex partner(s) may have HIV like you.
- Your sex partner(s) may not have HIV-even if you have been having sex for a long time.

⏩ Have your sex partner(s) and children been tested for HIV in the past 3 months?

If NO:

Your partner(s) and children should be tested for HIV

If YES:

- If your partner(s) are HIV-negative, they should be re-tested every 3 months.
- If your male partner(s) are HIV-negative, they should consider being circumcised.
- If your partner(s) or children have HIV, they need to get into care. Encourage them to come to the clinic and see a provider.

⏩ Have you disclosed to your sex partner(s)?

If NO:

- If you feel safe, tell your partner(s) that you have HIV. This may help them decide to get tested.

<u>INFORM</u> all clients on safer sex behaviours:

- Use lower risk practices such as mutual masturbation, oral sex, or thigh sex.
- Try to reduce your number of partners
- Use condoms every time you have vaginal or anal sex.

<u>If YES to any of the above:</u>
<u>INFORM</u> clients reporting higher risk behaviour:

- Use a condom for vaginal and anal sex with every sex partner.
- Use condom-compatible lubricant, especially for anal sex.
- If your partner refuses to use a condom, use lower risk practices such as mutual masturbation, oral sex, or thigh sex.
- Limit drinking or drug use before having sex.

<u>INFORM</u> client that there are negative consequences of having sex without a condom:

- You could give HIV to your HIV-negative partner(s).
- You or your sex partner(s) could give each other sexually transmitted infections.
- All of this can happen even if you are on medicines for HIV.
- If you/your partner(s) become pregnant you can pass HIV to the baby.

<u>ASSESS</u> client for alcohol or substance use that may affect adherence or risky behaviour:

- How many days a week do you drink alcoholic beverages such as beer or wine?
- In the last 12 months have you used illegal drugs (such as tik, nyaope, dagga, cocaine, heroin, speed)?

<u>INFORM</u> person that alcohol and other drug use can lead to faster disease progression, toxicity due to mixing alcohol and medications, and impaired decision making that can lead to unsafe sex. Refer or counsel patients on safer drinking or drug use behaviours such as limiting alcohol and drug use, especially before sex.

STEP 2: ASSESS client's adherence to ARVs and/or other medications.

- Do you take any medication for HIV?
- How many times did you miss a dose in the past week?
- What were the reasons for missing a dose?

<u>INFORM</u> client that HIV medication can greatly improve their health and prevent HIV transmission to uninfected partners and children. Provide or refer for adherence support, if needed.

STEP 3: ASSESS client for signs and symptoms of STIs.

- Do you have any: Abnormal penile/vaginal discharge? Pain on urination? Sores on your vagina, penis, or anus? Lower abdominal pain?

If YES:

- Identify syndrome and treat as indicated based on national guidelines.

<u>INFORM</u> client that their partner(s) should be notified and come for treatment.

STEP 4: ASSESS pregnancy status and intentions of patient/patient's partner

- Are you or your partner(s) trying to have a baby?

If YES:

- Provide family planning counselling and services for planning safe pregnancies including referral to ART services.

If NO:

- Refer client for or give contraception to prevent pregnancies.

<u>INFORM</u> client about the importance of planning pregnancies in HIV-positive women to reduce HIV transmission from mother to child.

STEP 5: GIVE client condom demonstration and condoms.

<u>INFORM</u> client that using condoms for every sex act help prevent HIV transmission.

ENDING VISIT:

⏩ Agree on a prevention goal for client to address and refer to a counsellor for further discussion (if available). Refer to community services for additional support if available.
