# Supplementary material for: Pre/post evaluation of a pilot prevention with positives training program for healthcare providers in North West Province, Republic of South Africa
Source: BMC Health Serv Res. 2017 May 2;17:316. doi: 10.1186/s12913-017-2263-7 (PMC5414361; doi:10.1186/s12913-017-2263-7)
Supplement: Supplementary file 2 — HIV Positive Patient Exit Questionnaire. This is the questionnaire that was designed for our study and completed by HIV positive patients at health facilities. (DOCX 41 kb) [file 12913_2017_2263_MOESM2_ESM.docx]

**Facility Number ___/___/___ Date___/___/___**

**Client Exit Interviews – HIV positive sequence**

**Section 1. Let’s begin with a few questions about you**

**1. My age is:** ______ **years**

**2. My ethnicity is: (Check all that apply):**

□Black

□White

□Asian

□Coloured

□Other [No additional response required.]

**3. I am a :**

□Male

□Female

□ Other

**4. The highest level of schooling that I completed is?**

□No school

□Some primary

□Completed primary

□Some secondary school

□Completed secondary school

□College, university or technikon

□Other (e.g. ABET)

**Section 2. The next questions are about visiting the health care facility today and your health**

**5.When I visited the facility today I saw (check all that apply):**

□Nurse

□Counsellor

□ Doctor

□ Other: ______________

**6. During my visit today, did the health care worker offer you an HIV test?**

□Yes

□No

□Decline to answer question

**7. Are you prepared to share your HIV status?**

□Yes , I am prepared to share my HIV status

□No, I’m not prepared to [Go to Section 2 of questionnaire for people who are HIV positive.]

□I don’t want to [Go to Section 2 of questionnaire for people who are HIV positive.]

**8. Do you know your HIV status?**

□Yes, I know my HIV status

□No, I don’t know my HIV status [Go to Section 2 of questionnaire for people who are HIV positive.]

□Decline

**9. What is your HIV status?**

□ I am HIV Positive

□I am HIV Negative [Go to Section 2 of questionnaire for people who are HIV positive.]

□ I don’t know my HIV status [Go to Section 2 of questionnaire for people who are HIV positive.]

□Decline [Go to Section 2 of questionnaire for people who are HIV positive.]

**10. I was diagnosed with HIV in: month/year**

__________(month) __/______(year)

**Section 3: Now some of the questions will be about visiting the health care facility today, and some will be about you.**

**For the questions about health care facility [the background is white]. For the questions about you, [the background is shaded in light green].**

|  | **Yes** | **No** | **Don’t know** | **De-cline** |
| --- | --- | --- | --- | --- |
| **Step 1 Prevention Recommendations** |  |  |  |  |
| 1. 1.0 During my visit today the Health Care Worker asked me about my sexual activity. |  |  |  |  |
| 1. 2.0 I have had sex within the last 3 months. |  |  |  |  |
|  |  | Skip to 5.0 |  |  |
| During my visit today, the health care worker told me that: |  |  |  |  |
| 2.1 My sex partner(s) may have HIV like me. |  |  |  |  |
| 2.2 My sex partner(s) may not have HIV-even if we have been having sex for a long time. |  |  |  |  |
| 1. 3.0 During my visit today, the health care worker asked me if my sex partners and children have been tested for HIV in the past 3 months. |  |  |  |  |
| 1. 4.0 My main sex partner been tested for HIV within the past 3 months. |  |  |  |  |
|  | Skip to 4.2 |  |  |  |
| 4.1 [If not tested] During my visit today, the health worker told me, my partner should be tested for HIV. |  |  |  |  |
|  | Skip to 5.0 | |  |  |
| 4.2 [If tested] I know my main partner’s HIV status. |  |  |  |  |
|  |  | Skip to 5.0 | |  |
| 4.3 [If known] My main partner is HIV positive. |  |  |  |  |
|  | Skip to 4.7 |  |  |  |
| 4.4 [If HIV negative] During my visit today the health worker told me my partner should be retested every 3 months? |  |  |  |  |
| 4.5 [If HIV negative] My main partner is male |  |  |  |  |
|  |  | Skip to 5.0 |  |  |
| 4.6 [If HIV negative and main partner is male] During the visit today, the health worker told me that my partner should consider being circumcised. |  |  |  |  |
|  |  | Skip to 5.0 |  |  |
| [If HIV positive] During my visit today, the health care worker told me: |  |  |  |  |
| 4.7 My partner should get care |  |  |  |  |
| 4.8 My partner should come to the clinic to see the provider |  |  |  |  |
| 5.0 I have children. |  |  |  |  |
|  |  | Skip to 7.0 |  |  |
| 6.0 [If yes] My youngest child been tested for HIV within the past 3 months. |  |  |  |  |
|  | Skip to 6.2 |  |  |  |
| 6.1 [If not tested] During my visit today, the health worker told me my youngest child should be tested for HIV. |  |  |  |  |
|  |  | Skip to 7.0 |  |  |
| 6.2 [If tested] I know my child’s HIV status. |  |  |  |  |
|  |  | Skip to 7.0 |  |  |
| 6.3 [If known] S/he HIV is positive. |  |  |  |  |
|  |  | Skip to 7.0 |  |  |
| [If HIV positive] During my visit today, the health care worker told me: |  |  |  |  |
| 6.4 My child should get care. |  |  |  |  |
| 6.5 My child should come to the clinic to see the provider. |  |  |  |  |
| 7.0 During my visit today, the health worker asked whether I disclosed my HIV status to my partner(s). |  |  |  |  |
| 8.0 I have disclosed my HIV status to my main partner. |  |  |  |  |
|  | Skip to 9.1 |  |  |  |
| [If not disclosed] During my visit today, the health worker |  |  |  |  |
| 8.1 Ask me if I would feel safe, telling my main partner that I have HIV. |  |  |  |  |
| 8.2 Told me that telling my main partner may help him/her decide to get tested. |  |  |  |  |
| During my visit today, the health worker told me about the following safer sex practices: |  |  |  |  |
| 9.1 Using low risk practices like mutual masturbation, oral sex, or thigh sex. |  |  |  |  |
| 9.2 Reducing the number of sex partners. |  |  |  |  |
| 9.3 Using condoms every time I have vaginal or anal sex. |  |  |  |  |
| During my visit today, the health worker told me about the consequences of having sex without condoms, such as: |  |  |  |  |
| 10.1 I could give HIV to my HIV-negative partner(s). |  |  |  |  |
| 10.2 My sex partner(s) and I could give each other sexually transmitted infections. |  |  |  |  |
| 10.3 All of this could happen even if I am on medications for HIV. |  |  |  |  |
| 10.4 If me or my partner become pregnant we can pass HIV to the baby. |  |  |  |  |
| During my visit today, the health worker asked me: |  |  |  |  |
| 11.1 How many days a week do I drink alcoholic beverages such as beer or wine. |  |  |  |  |
| 11.2 If I used illegal drugs in the last 12 months. |  |  |  |  |
| The health worker informed me that alcohol and other drug use can lead to: |  |  |  |  |
| 12.1 HIV progressing faster |  |  |  |  |
| 12.2 Toxicity due to mixing alcohol and medications |  |  |  |  |
| 12.3 Poor decision making that can lead to unsafe sex. |  |  |  |  |
| 13.1 How many days a week do you drink alcoholic beverages such as beer or wine? |  | |  |  |
| 13.2 In the last 12 months have you used illegal drugs? |  |  |  |  |
| 13.3[Does patient meet criteria for 13.1 > 0 or yes to 13.2?] |  |  |  |  |
|  |  | Skip to 14.1 |  |  |
| During my visit today, the health worker: |  |  |  |  |
| 13.4 Referred me for counselling. |  |  |  |  |
| 13.5 Counselled me on limiting alcohol or drug use. |  |  |  |  |
| 13.6 Counselled me about limiting alcohol or drug use before sex. |  |  |  |  |
| **Step 2. Assessing adherence to ARVs or other medications** |  |  |  |  |
| 14.0 During my visit today, the health worker asked me if I take medications for HIV? |  |  |  |  |
| 15.1 I am taking medications for HIV. |  |  |  |  |
|  |  | Skip to 16.1 |  |  |
| 15.2[If yes] I am currently on ART. |  |  |  |  |
|  |  | Skip to 16.1 |  |  |
| [If yes] During my visit today the health worker asked: |  |  |  |  |
| 15.3 How many times I missed a dose of my ARV’s in the past week. |  |  |  |  |
| 15.4 What were the reasons for missing a dose. |  |  |  |  |
| During my visit today, the health worker told me that HIV medication can |  |  |  |  |
| 16.1 Improve my health. |  |  |  |  |
| 16.2 Prevent HIV transmission to uninfected partners. |  |  |  |  |
| 16.3 Prevent HIV transmission to uninfected children. |  |  |  |  |
| **Step 3. Assess for signs and symptoms of STIs** |  |  |  |  |
| During my visit today the health worker asked if I have the following” |  |  |  |  |
| 17.1 Abnormal penile/vaginal discharge |  |  |  |  |
| 17.2 Pain on urination |  |  |  |  |
| 17.3 Sores on my vagina/penis or anus |  |  |  |  |
| 17.4 Lower abdominal pain |  |  |  |  |
| 18.0 I have at least one of the four symptoms from four previous four questions. |  |  |  |  |
|  |  | Skip to19.1 |  |  |
| [If yes to at least one] During my visit today the health worker |  |  |  |  |
| 18.1 Gave me treatment |  |  |  |  |
| 18.2 Told me to notify my partner |  |  |  |  |
| 18.2 Said my partner should come for treatment |  |  |  |  |
| **Step 4 Assess pregnancy status and intentions** |  |  |  |  |
| 19.1[If female] I am currently pregnant |  |  |  |  |
|  | Skip to 21.1 |  |  |  |
| During my visit today, did the health worker: |  |  |  |  |
| 19.2 Asked if my partner and I are trying to have a baby |  |  |  |  |
| 19.3 Informed me about the importance of planning pregnancies to reduce HIV transmission from mother to child |  |  |  |  |
| 20.0 My partner and I are trying to have a baby |  |  |  |  |
|  |  | Skip to 20.4 |  |  |
| [Yes, trying] Did the health worker: |  |  |  |  |
| 20.1 Provided family planning counselling |  |  |  |  |
| 20.2 Provided services for planning safe pregnancies |  |  |  |  |
| 20.3 Provided a referral for ART services |  |  |  |  |
|  | Skip to 21.1 | |  |  |
| [Not trying] Did the health worker: |  |  |  |  |
| 20.4 Referred me for contraception to prevent pregnancies |  |  |  |  |
| 20.5 Gave me contraception to prevent pregnancies |  |  |  |  |
| **Step 5 Give condom demonstration and condoms** |  |  |  |  |
| During my visit today the health worker: |  |  |  |  |
| 21.1 Gave me a condom demonstration |  |  |  |  |
| 21.2 [If female] gave me a female condom demonstration |  |  |  |  |
| 21.3 Gave me condoms |  |  |  |  |
| 21.4 [If female] gave me female condoms |  |  |  |  |
| 21.5 Told me that using a condom for every sex act helps prevent HIV transmission |  |  |  |  |
| 22.1 During my visit today, the health worker agree on a prevention goal: |  |  |  |  |
|  |  | Skip to 23.1 |  |  |
| 22.2 [If agreed on goal] My prevention goal is. |  | | | |
| During the visit today, the health worker: |  |  |  |  |
| 23.1 Referred me to a counsellor for further discussion |  |  |  |  |
| 23.2 Referred me to community services for additional support |  |  |  |  |

Thank you for your time and responses. We hope the information will help improve the quality of care at facilities in this district. Thanks, again.
